# Towards a molecular understanding of symbiont function: Identification of a fungal gene for the degradation of xylan in the fungus gardens of leaf-cutting ants

**DOI:** 10.1186/1471-2180-8-40

**Published:** 2008-02-28

**Authors:** Morten Schiøtt, Henrik H De Fine Licht, Lene Lange, Jacobus J Boomsma

**Affiliations:** 1 Department of Biology, University of Copenhagen, Universitetsparken 15, 2100 Copenhagen, Denmark; 2 Department of Molecular Biology, University of Copenhagen, Ole Maaløes vej 5, DK-2200 Copenhagen, Denmark

## Abstract

**Background:**

Leaf-cutting ants live in symbiosis with a fungus that they rear for food by providing it with live plant material. Until recently the fungus' main inferred function was to make otherwise inaccessible cell wall degradation products available to the ants, but new studies have shed doubt on this idea. To provide evidence for the cell wall degrading capacity of the attine ant symbiont, we designed PCR primers from conserved regions of known xylanase genes, to be used in PCR with genomic DNA from the symbiont as template. We also measured xylanase, cellulase and proteinase activities in the fungus gardens in order to investigate the dynamics of degradation activities.

**Results:**

We cloned a xylanase gene from the mutualistic fungus of *Acromyrmex echinatior*, determined its protein sequence, and inserted it in a yeast expression vector to confirm its substrate specificity. Our results show that the fungus has a functional xylanase gene. We also show by lab experiments *in vivo *that the activity of fungal xylanase and cellulase is not evenly distributed, but concentrated in the lower layer of fungus gardens, with only modest activity in the middle layer where gongylidia are produced and intermediate activity in the newly established top layer. This vertical distribution appears to be negatively correlated with the concentration of glucose, which indicates a directly regulating role of glucose, as has been found in other fungi and has been previously suggested for the ant fungal symbiont.

**Conclusion:**

The mutualistic fungus of *Acromyrmex echinatior *has a functional xylanase gene and is thus presumably able to at least partially degrade the cell walls of leaves. This finding supports a saprotrophic origin of the fungal symbiont. The observed distribution of enzyme activity leads us to propose that leaf-substrate degradation in fungus gardens is a multi-step process comparable to normal biodegradation of organic matter in soil ecosystems, but with the crucial difference that a single fungal symbiont realizes most of the steps that are normally provided by a series of microorganisms that colonize fallen leaves in a distinct succession.

## Background

**Figure 1:**
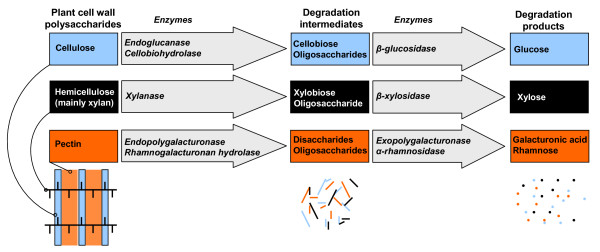
**Plant cell wall degradation**. Schematic overview of the structure of a plant cell wall and the most important enzymatic reactions involved in the degradation of its polysaccharides. Cellulose microfibrils (blue) are cross-linked by hemicellulose chains (black) within a matrix of pectin (orange). The complex polysaccharides are degraded to disaccharides and oligosaccharides, which are further degraded to soluble monosaccharides that can be assimilated. Full degradation of hemicellulose and pectin may involve more enzymes than those presented here (up to ca. 17 for hemicellulose and ca. 24 for pectin [23]).

Neo-tropical leaf-cutting ants of the genera *Acromyrmex *and *Atta *live in symbiosis with the basidiomycete fungus *Leucoagaricus gongylophorus*, which they rear in underground gardens and provide with fresh leaf material. This mutualistic interaction provides the ants with easily digested food in the form of specialized nutrient-rich hyphal tips, the gongylidia. It has been assumed that the ants obtain major benefits from the enzymatic capacity of the fungus to degrade polysaccharides from plant cell walls [1-3], but this view has recently been challenged by findings that *L. gongylophorus *grows only poorly on synthetic media containing cellulose [4,5]. Plant cell walls consist mainly of polysaccharides in the form of cellulose microfibrils, embedded in a matrix of hemicellulose and pectin (fig. 1). Cell wall material accounts for 30–50 % of leaf dry mass [6], so the ability of the mutualistic fungus to utilize cell walls has a major influence on the amount of foliage needed to sustain a leaf-cutting ant colony. Likewise, the extent of degradation of the leaf material harvested by the ants will determine the amount of fungal waste that colonies have to process to avoid infectious diseases [7,8] and the extent to which these waste products are the origin of a further decomposition food-chain. The value of New World crops destroyed by leaf-cutting ants each year is counted in billions of dollars [3], so that the clarification of functional questions on the degradation of cell wall material has both significant economical and ecological relevance.

During evolution, the degradation of cell wall material became a particularly acute resource problem for the fungus-growing ants when they started to solely use fresh leaves as substrate for their fungus gardens. This happened in the common ancestor of the *Atta *and *Acromyrmex *leaf-cutting ants and coincided with an entire suite of other transitions towards large colony size, substantial worker caste differentiation and higher genetic diversity via multiple queen mating [9]. Large colonies with high turnover rates are more likely to be resource-constrained and are more vulnerable to pathogen infections, in particular when they accumulate large amounts of waste [10]. Any ability of the fungal symbiont to degrade cell wall material would imply more efficient resource acquisition and less waste, so that documenting such abilities would contribute to our general understanding of the evolutionary and ecological success of the fungus-growing ants.

A major constituent of plant cell walls is hemicellulose. Most of this is xylan, consisting of a backbone of xylose molecules linked by 1,4-β-xylose residues, to which side groups of 4-*O*-methyl-D-glucuronopyranosyl, α-L-arabinofuranosyl, acetyl, feruloyl, and *p*-coumaroyl can be coupled [11]. Degradation of the xylan backbone is catalyzed by endo-1,4-β-xylanases (EC 3.2.1.8) that cleave the backbone randomly into xylo-oligosides, and by β-D-xylosidases (EC 3.2.1.37) that split off xylose monomers from the non-reducing end of xylo-oligosides (fig. 1). Based on sequence similarity, the fungal endoxylanases fall into three groups belonging to, respectively, the glycoside hydrolase families 5 (rarely), 10 and 11 [12,13]. Most of the ca. 100 endoxylanases isolated so far originate from Ascomycota: a search in the Carbohydrate-Active Enzymes database [14,15] produced only six basidiomycete endoxylanase sequences in glycoside hydrolase family 10 and four in glycoside hydrolase family 11 for which the substrate specificity has been verified.

The objective of the present study was to directly assess the presence and functional activity of endoxylanases in the ant-cultivated fungus *Leucoagaricus gongylophorus *by cloning an endoxylanase gene and confirming its substrate specificity by heterologous expression in yeast cells. We also directly assayed endoxylanase activity in live fungus garden material of *Acromyrmex echinatior *and show that the activity is highest in the oldest parts of fungus gardens where the concentration of glucose is very low.

## Results

**Figure 2:**
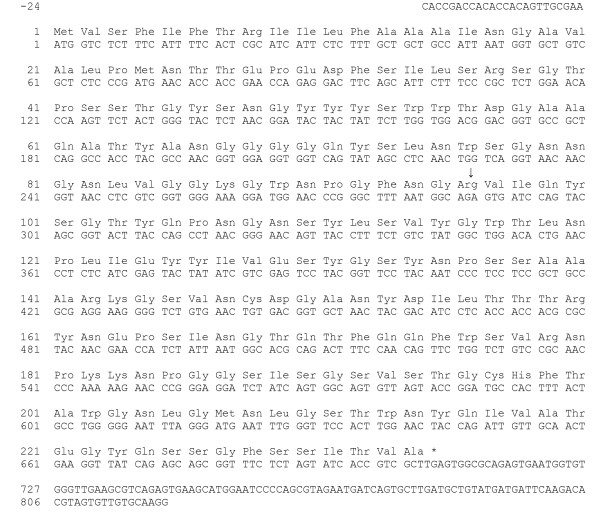
**Xylanase sequence**. cDNA and protein sequence of the LgXyn1 xylanase gene in the *Leucoagaricus gongylophorus *fungal symbiont of the leaf-cutting ant *Acromyrmex echinatior*. Numbering of amino acids and nucleotides starts from the start codon (ATG). The position of the single intron of this gene is indicated by an arrow, and the stop codon is indicated by an asterisk.

Degenerate primers designed from conserved domains of known fungal xylanases amplified a gene (LgXyn1) [GenBank:EF208066] with very high similarity to fungal xylanases belonging to glycoside hydrolase family 11 [12,13]. The rest of the gene sequence was obtained with a RACE (Rapid Amplification of cDNA Ends) based method, using specific primers designed from the initially amplified sequence. The protein sequence (fig. 2) showed 78 % amino acid identity (Blast score = 251 bits, E-value = 2e^-65^; 196 of the 234 amino acids) to a xylanase from the basidiomycete *Schizophyllum commune *[16] and 61 % identity (Blast score = 221 bits, e-value = 3e^-56^; 198 of the 234 amino acids) to a xylanase from the ascomycete *Thermomyces lanuginosus *[17]. The 5' untranslated region of the cDNA transcript is only 24 bp long, whereas the 3' untranslated region is 120 bp. The gene has an intron of 56 base pairs positioned between nucleotide 287 and 288 after the start codon (fig. 2).

**Figure 3:**
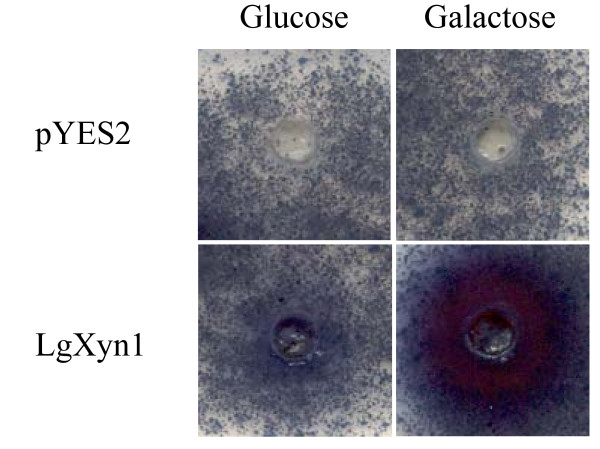
**Heterologous expression of xylanase gene**. Activity plate assays with Azur-linked Xylan (AZCL, Megazyme). The two top panels show assays using extracts of yeast cells transformed with an empty vector (pYES2), which was expected to give no reaction. The two bottom panels show assays using extracts of yeast cells transformed with cDNA of LgXyn1 inserted in pYES2. These show a clear indication of xylanase activity, with the strongest activity being observed when the yeast cells were grown on galactose-containing media, which induces transcription of the inserted cDNA.

The xylanase cDNA sequence was inserted in the yeast expression vector pYES2 downstream of the galactose inducible promoter, and transformed into yeast cells. Extracts from transformed yeast cells grown on medium containing galactose showed strong xylanase activity when tested on AZCL-assay plates, while yeast cells grown on medium containing glucose showed only weak xylanase activity (fig. 3), presumably because the promoter has some residual activity even in the absence of galactose. Yeast cells transformed with empty vector showed no xylanase activity, neither on galactose nor on glucose containing medium (fig. 3).

Stable fungus gardens of leaf-cutting ants have three discernible layers. The top layer has a high proportion of newly incorporated tiny leaf fragments that the ants recently placed on the upper ridges of the garden. The top layer is therefore normally characterized by its darker pigmentation as the newly grown hyphae have not yet degraded the chlorophyll of the leaf material. The middle layer is more compact and completely white, and has a high density of swollen hyphal tips, the so-called gongylidia. The bottom layer is the oldest part of the garden, has a somewhat darker and drier appearance and has fewer gongylidia. This stratification of the fungus garden implies that mycelium and substrate are continuously moving downward in a fungus garden, a process that takes ca. 6 weeks to be completed in our *Acromyrmex *lab colonies. Our laboratory rearing technique with fungus-gardens under inverted 1 l beakers [18] accurately mimics the field situation in which fungus chambers have a similar size and shape and where the same differently colored layers can often be observed.

**Figure 4:**
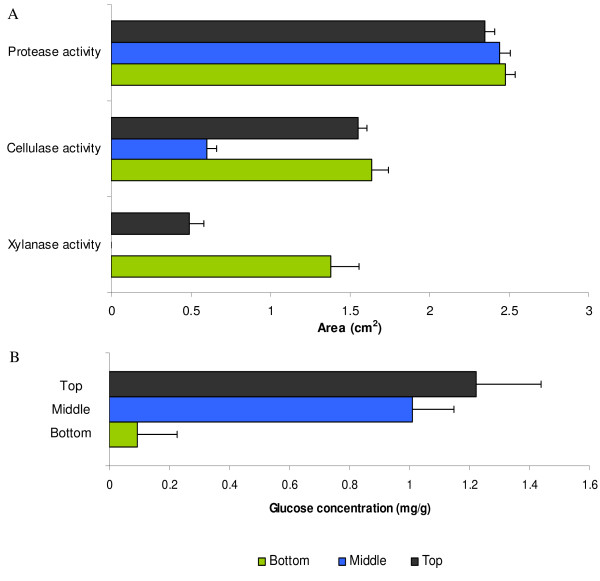
**Enzyme activities and glucose concentration infungus gardens**. A, enzyme activities in samples of fungus garden material from the top, middle or bottom layer o f the fungus gardens of three *A. echinatior *colonies (Ae150, Ae219, Ae322) measured as the area of the blue halo 22 hours after incubation on AZCL-casein, AZCL-HE-cellulose or AZCL-xylan (see B-panel for column identities). B, glucose concentration in the top, middle and bottom layer of the fungus garden of colony Ae322 in mg glucose per g fungus garden. All error bars are SEs.

Whereas protease activity was distributed evenly from bottom to top in the three fungus gardens tested (Kruskall-Wallis, χ^2 ^= 2.15, df = 2, *p *= 0.342), xylanase activity was strongest at the bottom, only weak at the top and completely absent in the middle layer (Wilcoxon top-bottom, *Z *= 3.47, *p *= 0.0005) (fig. 4A). The activity pattern of cellulase resembled that of xylanase, although stronger activity was observed at the top and in the middle layer (Wilcoxon top-bottom, *Z *= 0.28, *p *= 0.779; Wilcoxon top-middle, *Z *= -6.22, *p *< 0.001; Wilcoxon middle-bottom, *Z *= -5.95, *p *< 0.001) (fig. 4A).

In bacteria and Ascomycota, glucose has been shown to suppress the expression of xylanase and cellulase [19-23]. We therefore investigated whether the layer-specific activity of xylanase and cellulase in gardens of *Leucoagaricus gongylophorus *were related to glucose concentrations. We measured glucose concentrations in 10 samples for each of the three layers of the fungus garden of colony Ae-322 and found that the top and middle layer contained about ten times as much glucose than the bottom layer (Wilcoxon top-middle, *Z *= -0.4536, *p *= 0.6501; Wilcoxon top-bottom, Z = -3.7796, *p = *0.0002; Wilcoxon middle-bottom, Z = 3.7041, *p *= 0.0002; fig. 4B).

## Discussion

In the present study we have cloned a xylanase gene from DNA and mRNA extracted from *Acromyrmex echinatior *fungus-garden material, and we have amplified the same gene from plated monocultures of the fungal symbiont. These findings shed important light on the ongoing debate whether the fungal symbiont of leaf-cutting ants has evolved from saprophytic or biotrophic free living ancestors [24,5]. Saprophytic fungi produce extracellular enzymes to degrade complex organic compounds such as plant cell walls, whereas biotrophic fungi lack most of the enzymes needed to degrade plant cell walls, but are able to break down pectin to gain access to living plant cells [5]. Our present finding that the *Leucoagaricus gongylophorus *symbiont of *A. echinatior *has an expressed gene encoding a xylanase is thus consistent with a saprotrophic origin of the fungal symbiont of attine ants. This also matches the phylogenetic evidence showing that the gongylidia-producing symbionts of the higher attines are a derived group of fungi originating from an ancestor that was likely cultivated by a lower attine ant, where it used dead but not highly degraded plant material [25-27]. Although the fungal symbiont has the enzymatic capacity to degrade xylan, it is as yet unknown whether the fungus is also able to further degrade the xylobiose product into xylose, and whether the fungus can assimilate xylose and use it as a carbon source (fig. 1). The ants, however, are able to survive on xylose and therefore likely able to utilize xylose as a carbon source [28]. An alternative hypothesis is that the fungus produces xylanase merely to break down hemicellulose in the cell walls of leaf material to gain access to the easy degradable compounds within the cells but without assimilating the degradation products of xylan. However, as biotrophic fungi apparently are able to penetrate the cell wall without the need of cell wall degrading enzymes, we don't find this alternative hypothesis plausible.

Previous studies have measured xylanase activity in the fungus gardens of leaf-cutting ants, either by determining enzyme activity in fungal material taken directly from gardens [29,30] or from symbiont fungus grown *in vitro *on xylan-containing medium [31]. However, the fungus gardens of leaf-cutting ants are home to a wide array of microorganisms some of which have polysaccharidase activity [32-39]. It can therefore not be ruled out that some of the enzymatic activities found in earlier studies might have originated from these other microorganisms or from ants or substrate material incorporated in the garden. Our present molecular approach resolves this ambiguity and allows us to conclude that the fungal symbiont has the enzymatic capability to degrade xylan, and to make the inference that at least a large part of the xylanase activity observed in this study and in previous studies derives from the fungal symbiont of the ants. This also underlines that our approach has considerable potential for studying the activity of other enzymes in attine fungus gardens.

### Fungus gardens as natural decomposition gradients

The observed patterns of enzyme activities in the fungus gardens suggests that degradation of plant tissue takes place as a multi-step process, starting at the top of the garden where new substrate is applied by the ants, and ending at the bottom of the garden with old substrate that eventually will be discarded by the ants. The process starts with the degradation of compounds with high nutritious value, such as proteins that have a high content of nitrogen, and ends with the degradation of plant cell wall polysaccharides, which can be assumed to have a lower nutritious value. A multi-step degradation process of leaf substrate in fungus gardens is appealing as a working hypothesis, because it would match the general pattern of biodegradation of organic matter in soil ecosystems, where specialized decomposers colonize the dead plant material in a distinct succession, starting with fast growing populations of bacteria and so-called sugar fungi that assimilate the readily available soluble compounds [40-42], followed by slower growing ascomycete and basidiomycete fungi that are able to degrade the structural components of the plant material.

Our present results do not allow us to establish that the measured enzyme activities in fungus gardens are due to the activity of only a single symbiont, *Leucoagaricus gongylophorus*, or whether multiple microorganisms are involved, but we expect that the possible contributions of other microorganisms have been minor, given the massive dominance of biomass of the fungal symbiont. However, in either scenario the ants can potentially regulate the efficiency of degradation by adjusting the length of time the substrate is staying in the garden, and thereby regulate the release of nutrients according to the actual nutritional need of the colony.

In previous studies of xylanase and cellulase activity in the fungus gardens of *A. echinatior *or closely related leaf-cutting ant species, a large variation in enzyme activity was found [28-31]. The levels of xylanase activity found in these studies range from 32 μg hp/m/gww (hydrolysis products per min per gram wet weight of fungal cells) [30] to 567 μmol hp/m/gdw (hydrolysis products per min per gram dry weight of fungal cells) [28], and the levels of cellulase activity range from 7.5 μg hp/m/gww [30] to 646 μmol hp/m/gdw [28]. In both cases this corresponds to a difference of about three orders of magnitude. In contrast our analyses had relatively modest SE's around the averages obtained. We therefore hypothesize that at least part of this earlier variation might be explainable by our finding that xylanase and cellulase activity is not uniform within fungus gardens, but predominantly concentrated in the oldest parts.

### Molecular and behavioural mechanisms maintaining stratified fungus gardens

Many xylanolytic and cellulolytic genes are co-regulated in microorganisms, which seems logical because the natural degradation of cellulose and hemicellulose is normally tightly connected. Both types of enzymes are often observed to be induced by D-xylose, xylobiose, xylan, cellobiose and cellulose and to be repressed by glucose [19-23]. Induction of these genes seems to be mediated by the transcription activator XlnR, which binds to conserved motifs in the promoter region of cellulolytic and xylanolytic genes to induce expression [21,23]. Repression, on the other hand, is mediated by the transcription repressor protein CreA (a protein isolated form *Aspergillus*) or Cre1 (the ortholog of CreA isolated from *Trichoderma*) [22,23,43-45]. Like XlnR, CreA/Cre1 binds to a conserved motif in the promoter region of the gene, but in this case transcription is inhibited. We hypothesize that similar molecular mechanisms are governing the expression of degradation enzymes in the fungus gardens of leaf-cutting ants, which may explain the observed correlation of cellulase and xylanase activities in the middle and lower parts of the gardens (fig. 4A) and their inverse relationship with glucose concentrations. A role of glucose as a regulator of the expression of polysaccharide degrading enzymes has also been suggested by Silva et al. [46,47], and is in agreement with findings in other filamentous fungi, where glucose is known to be a repressor of xylanolytic and cellulolytic enzyme genes. This effect of glucose would be logical as there is no need to produce polysccharide degrading enzymes when glucose is abundant, whereas a low glucose concentration would indicate a low level of monosaccharides and the need to degrade polysaccharides.

In the top layer we did not find this correlation between glucose levels and enzyme activities, as glucose levels were comparable to those in the middle layer, whereas xylanase activity was intermediate and cellulase activity was high. One possible explanation could be that the top layer of a fungus garden also contains inducers of cellulase and xylanase expression that compensate for the repression effect of glucose. Another possibility is that the cellulase and xylanase activity in the top layer is in fact not produced by the top mycelium, as it has previously been shown that leaf-cutting ants mix new leaf material with fecal fluids before inserting the fragmented leaf material into the fungus garden [1]. The fecal droplets contain proteins originating from the fungal symbiont, and apparently pass undigested through the alimentary channel of the ants [48-53]. A broad spectrum of enzymes has been found in the fecal droplets, and among these are some that show xylanolytic and cellulolytic activity [29,30,50,52]. However, whether these enzymes originate from the ants, the symbiont food ingested by the ants, or another organism in the fungus garden remains to be unequivocally determined. Clarifying the molecular details of enzyme transfer via the fecal droplets may therefore shed very interesting light on the advances of fungus farming that the evolutionary derived leaf-cutting ants achieved relative to their ancestors who could, together with their fungal symbionts, only process dead organic substrate.

## Conclusion

The fungal symbiont of the leaf-cutting ant *Acromyrmex echinatior *expresses a functional xylanase gene, which indicates that it can degrade plant cell wall material and corroborates that the attine ant symbiont has a saprotrophic origin.

Enzymatic degradation activity varied considerably between different layers of fungus gardens suggesting that the degradation of leaf substrate is subdivided in a series of distinct enzymatic steps.

Cellulase and xylanase activities were to some extent negatively correlated with glucose concentrations, indicating that glucose could have a regulatory role for the expression of these enzymes.

## Methods

### Biological material

Colonies of *Acromyrmex echinatior *(numbers Ae150, Ae219, Ae300 and Ae322) were collected in Gamboa, Panama and maintained in the laboratory under standard conditions of 25°C and 70% relative humidity [18] where they were supplied with a diet of bramble leaves, rice and pieces of apple. Pure cultures of the symbiotic fungus were obtained by inoculating mycelium collected from fungus gardens onto potato dextrose agar (PDA) plates, and incubating them at 25°C in the dark. A characteristic of the fungal symbiont of higher attine ants is the formation of gongylidia [1,25,54]. The presence of gongylidia in the fungus cultures was thus used as an indicator for the isolation of the right species of fungus. Further proof of species identity was obtained by sequencing the ITS region from these isolates [55]. DNA was obtained by grinding fresh fungus material from fungal gardens and fungal cultures in liquid nitrogen and extracting DNA using a CTAB based method as described by Saghai-Maroof et al. [56] with small modifications. As our objective was to obtain cDNA and protein sequences, we also obtained total RNA from the same fungus garden material by grinding in liquid nitrogen and extracting RNA using the RNeasy Plant Mini Kit (QIAGEN).

### Cloning of LgXyn1

To obtain candidate xylanase genes, several degenerate primers were designed from conserved motifs in fungal xylanases, which were found by aligning known sequences of fungal xylanases belonging to glycoside hydrolase families 10 and 11. These primers were used in PCR to amplify the corresponding sequence from *Leucoagaricus gongylophorus *genomic DNA obtained from colony Ae300. The degenerate sense primer 5'-TTY GTN GGI GGN AAR GGI TGG-3' and the degenerate antisense primer 5'-CCY TCN GTI GCI CAN AYY TG-3' amplified a fragment of 475 bp using the following PCR scheme: one cycle of 95°C for 2 min, then 20 cycles of 95°C for 30 sec, 54°C for 30 sec and 72°C for 1 min, followed by 15 cycles of 95°C for 30 sec, 54°C for 30 sec and 72°C for 2 min, and ending with one cycle of 72°C for 7 min. From the amplified sequence obtained, specific forward and reverse primers were designed, which were used to clone the 5'end and 3'end using the SMART RACE cDNA kit (CLONTECH). The 3'end was cloned using the sense primer 5'-AAT TTA GGG ATG AAT TTG GGT TCC-3' with PCR conditions consisting of one cycle of 95°C for 3 min, then 12 cycles of 95°C for 30 sec, 70°C for 30 sec (with a 0.8°C decrease every cycle) and 72°C for 2 min, followed by 30 cycles of 95°C for 30 sec, 60°C for 30 sec and 72°C for 3 min., and ending with one cycle of 72°C for 10 min. The 5'end was cloned using the antisense primer 5'-ACC CCT TCC TCG CGG CAG CGG AGG AGG G-3', followed by the nested primer 5'-GTA AGT ACC GCT GTA CTG GAT CAC TCT GCC-3', which was designed to span the intron. The PCR conditions were as follows: one cycle of 95°C for 5 min, then 40 cycles of 95°C for 30 sec, 68°C for 30 sec and 72°C for 1 min with an extension of 2 sec every cycle, and ending with one cycle of 72°C for 7 min. All PCR products were cloned in pCR4-TOPO before sequencing using the TOPO TA cloning method (Invitrogen). The position and sequence of the intron was determined by alignment of the two gene sequences obtained from genomic DNA and cDNA. The xylanase gene was finally amplified and sequenced from DNA extracted from pure fungus cultures, to confirm its origin from the fungal symbiont.

### Heterologous expression in yeast

Heterologous expression of the xylanase cDNA sequence in *Saccharomyces cerevisiae *was done to confirm the xylanase identity of the protein and to determine whether the fungal xylanase sequence was functionally active. To this end, the coding sequence of the xylanase gene was PCR amplified from cDNA using the sense primer 5'-AAG CTT CAC CAC AGT TGC AGA ATG GTC-3' together with the antisense primer 5'-TCT AGA TCA AGC GAC GGT GAT ACT AG-3'. PCR conditions were one cycle of 95°C for 5 min, then 15 cycles of 95°C for 30 sec, 55°C for 30 sec and 72°C for 1 min, followed by 20 cycles of 95°C for 30 sec, 55°C for 30 sec and 72°C for 1 min with an extension of 2 sec every cycle, and ending with one cycle of 72°C for 7 min.

The amplified cDNA was sequenced, to ensure that no PCR errors had been incorporated into the construct, and inserted into the yeast expression vector pYES2 (Invitrogen) under the control of a galactose inducible promoter. *Saccharomyces cerevisiae *strain INVSc1 (*MATa his3D1 leu2 trp1-289 ura3-52*, Invitrogen) was transformed with either the pYES2 vector containing the xylanase gene (pYES2-LgXyn1) or an empty pYES2 vector using the LiOAc/polyethylene glycol (PEG) method [57]. As the pYES2 vector contains the *URA3 *gene, positive transformants could be selected by plating on a uracil depleted yeast medium consisting of 2 % glucose, 2 % bacto-agar, 0.7 % (wt/vol) yeast nitrogen base (Invitrogen) and 0.2 % yeast synthetic drop-out media supplement without uracil (Sigma-Aldrich), and incubating at 25°C for 3 days. Ura^+ ^colonies were grown in a similar medium without agar and with either 2 % glucose or 2 % galactose at 25°C over night. Yeast cells from 5 ml culture were harvested by centrifugation, ground in liquid nitrogen with a mortar and pestle, and dissolved in an equal amount of 50 mM Tris pH 7.0 to be used in an AZCL-xylan assay.

### AZCL plate assays

To investigate which parts of the fungus gardens of laboratory colonies expressed xylanase, cellulase and protease activity, an agarose medium consisting of 1% agarose, 23 mM phosphoric acid, 23 mM acetic acid and 23 mM boric acid of pH 4.7 was heated until the agarose was melted and then cooled to 65°C. After this, 0.1 % (w/v) AZCL-xylan, AZCL-HE-cellulose or AZCL-casein (Megazymes) was added and the medium was poured into petri dishes. After the medium had solidified, wells were made with a cut off pipette tip to give a diameter of ca. 4 mm. Fungus garden proteins were extracted by grinding ca 100 mg fresh fungal material with a sterile pestle in eppendorf tubes containing 2.5 times as much 50 mM Tris pH 7.0. The extract was centrifuged at 4°C for 15 min at 15,000 g, and 20 μl of the supernatant was applied to each well. After 22 hours of incubation at 25°C the plates were photographed and the area of the blue halo surrounding the well (which is a quantitative measure for the amount of substrate degraded) was measured using the software program ImageJ ver. 1.29w. Ten samples of 100 mg fungal material were taken from the top, middle and bottom layer of the gardens of three colonies (Ae150, Ae219 and Ae322). All these 90 samples were tested for xylanase, cellulase and protease activity using AZCL-xylan, AZCL-HE-cellulose and AZCL-casein respectively. Statistical tests were done with the statistical package S-plus version 6.1 for Windows.

### Glucose assay

To determine the glucose concentration in fungal garden material from the same top, middle and bottom parts of fungus gardens, extracts were prepared as described for the AZCL plate assays. The glucose concentration of the supernatant was measured using the Rotitest d-Glucose kit (Carl Roth) as described by the manufacturer with minor modifications. Briefly, 5 μl supernatant was mixed with 50 μl assay buffer and 95 μl water. The absorbance was then measured at 340 nm using a plate reader (Molecular Devices) before and after incubation with 1 μl Hexokinase/Glucose-6-Phosphate mixture. Calculation of glucose content was performed as described in the manual provided by the manufacturer. The glucose concentration was determined in 10 samples taken from each of the three layers of a single fungus garden (Ae322).

## Authors' contributions

JJB and LL conceived of the study. MS and HHDFL designed and carried out the experimental work. MS and JJB drafted the manuscript, and HHDFL and LL contributed to the final manuscript. All authors read and approved the final manuscript.
